# Evaluation of ablation index-guided linear lesions formation for left atrial arrhythmia treatment

**DOI:** 10.1007/s10840-025-02232-3

**Published:** 2026-02-11

**Authors:** Vanessa Sciacca, Sascha Hatahet, Christian-H. Heeger, Roman Mamaev, Egzona Pajaziti, Ly Nghiem, Julia Schulten-Baumer, Anna Traub, Samuel Reincke, Bettina Kirstein, Darko Trajanoski, Huong Lan Phan, Niels Große, Lisbeth Delgado Lopez, Julia Vogler, Hisaki Makimoto, Charlotte Eitel, Karl-Heinz Kuck, Thomas Fink, Roland Richard Tilz

**Affiliations:** 1 Department of Rhythmology, University Heart Center Lübeck, Ratzeburger Allee 160, Lübeck, 23538 Germany; 2 Department of Electrophysiology, Heart and Diabetes Centre North Rhine-Westphalia, Ruhr-University Bochum, Bochum, Bad Oeynhausen Germany; 3 German Center for Cardiovascular Research (DZHK), Partner Site Hamburg/Kiel/Lübeck, Lübeck, Germany; 4 Department of Cardiology, Angiology and Intensive Care Medicine, University Heart Center Lübeck, University Hospital Schleswig-Holstein, Lübeck, Germany; 5 LANS Cardio, Stephansplatz 5, 20354 Hamburg, Germany; 6 Data Science Center/Cardiovascular Center, Jichi Medical University, Tochigi, Japan; 7 Clinic for Electrophysiology Herz- und Diabeteszentruum NRW, Georgstraße 11, 32545 Bochum, Bad Oeyenhausen Germany

**Keywords:** Linear lesions ablation, Reconduction linear lesions, Ablation index, Contact force

## Abstract

**Background:**

Catheter based left atrial linear lesion ablation is commonly performed in patients with atrial fibrillation (AF) and complex left atrial substrate or in patients presenting with macro-reentrant left atrial tachycardia (LAT).

**Aims:**

To assess acute and long-term effectiveness, procedural viability and safety of ablation index (AI)-guided compared to contact force (CF)-guided ablation of left atrial linear lesions.

**Methods:**

Consecutive patients undergoing left atrial ablation for AF or LAT including left atrial linear lesions ablation either guided by AI or CF without AI were prospectively enrolled. Characteristics of anterior lines (AL), mitral isthmus lines (MIL), roof lines (RFL) and posterior wall isolation (PWI) were systematically analyzed in both cohorts. Procedural feasibility, safety, acute and long-term block of linear lesions as well as clinical outcome in terms of arrhythmia recurrence were assessed.

**Results:**

A total of 313 patients were included. 260 patients (83.1%) underwent AI-guided ablation and 53 patients (16.9%) CF-guided ablation. Complete conduction block was comparable between study groups (94 vs. 95% for AL, 94 vs. 95% for AL100 vs. 99% for RFL and 100 vs. 100% for PWI). Acute block of MIL was significantly more often achieved in AI-guided ablation (100 vs. 67%, *p* = 0.0004). No significant difference in persistence of conduction block for AL (*p* = 0.47), MIL (*p* = 0.57), RFL (*p* = 0.18) or PWI (*p* = 1) could be observed between AI-guided or CF-guided ablation during repeat ablation. Survival analysis showed no significant differences in estimated arrhythmia free survival (*p* = 0.45). Periprocedural complications occurred in 22 patients (7%) with equal distribution in CF-guided and AI-guided ablation (*p* = 0.31).

**Conclusion:**

AI-guided ablation is associated with significantly higher rates of acute conduction block across MIL, shorter RF times and lower FTI for AL and RFL while complication rates and long-term clinical outcome are comparable to non-AI-guided ablation.

**Graphical Abstract:**

**Figure:**
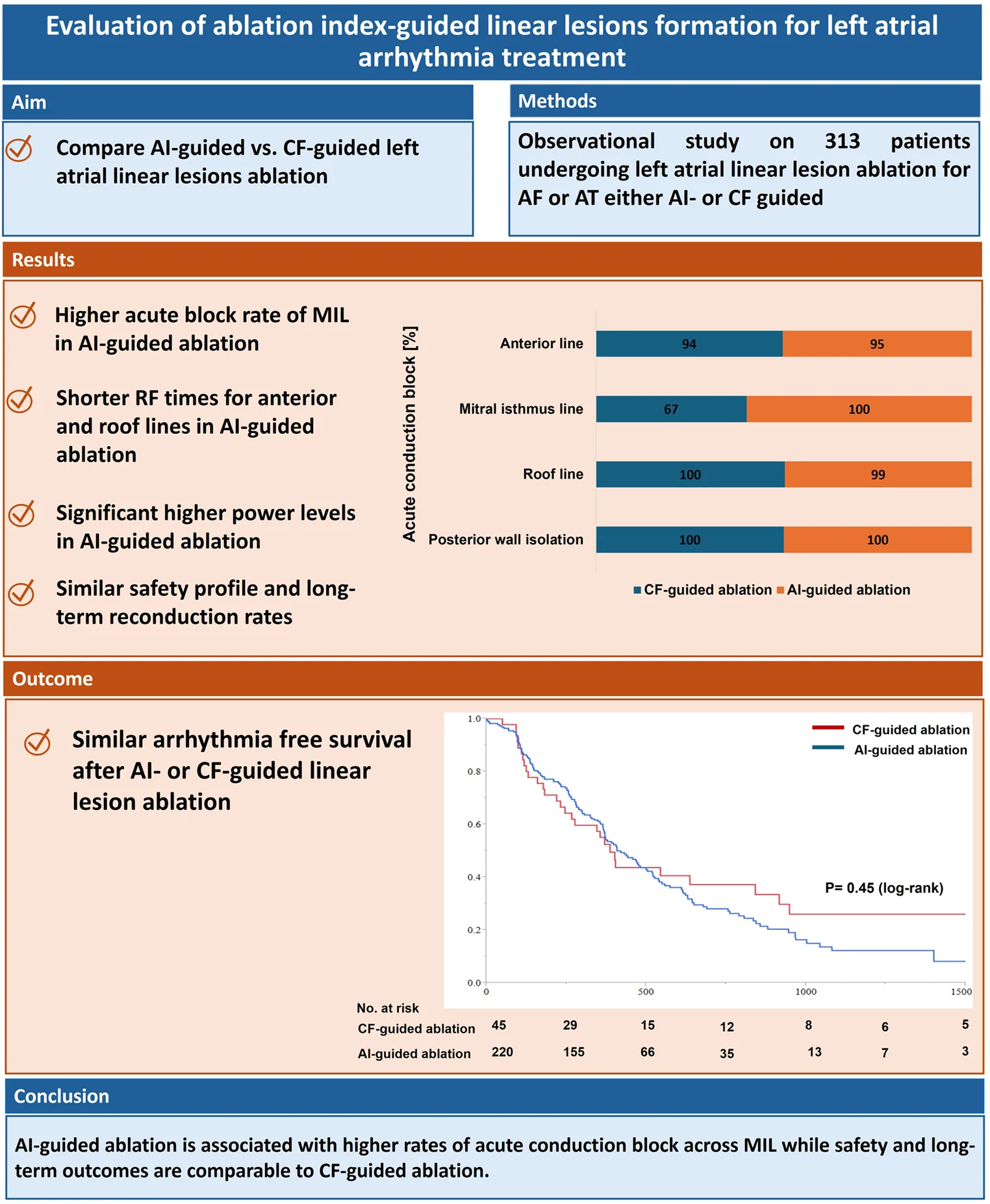

## Introduction

Catheter ablation has evolved an indispensable treatment for patients with atrial fibrillation (AF) and other atrial arrhythmias such as left atrial tachycardia (LAT). Pulmonary vein isolation (PVI) remains the cornerstone of AF ablation [1]. However, PVI alone may not be sufficient to provide long-term rhythm control in patients with persistent disease states and complex left atrial substrate or in patients presenting with LAT [2, 3]. Catheter based left atrial linear lesion deployment is commonly performed to treat AF substrate beyond PVI or to address macro-reentrant LAT. Left atrial compartmentalization, partial denervation, elimination of non-pulmonary vein triggers and prevention of macro-reentrant LAT are considered beneficial effects of linear lesions [4]. Durable conduction block along deployed linear lesions is mandatory to achieve long-term antiarrhythmic effects and to avoid proarrhythmic states [5]. However, deployment of fully blocked durable linear lesions may be challenging. Surrogates such as contact force (CF) or impedance drop are widely used to determine lesion quality during radiofrequency (RF) ablation. Ablation index (AI) (CARTO 3, Biosense Webster, Inc., Diamond Bar, CA) is a marker of RF lesion quality incorporating CF, ablation time, catheter stability and power in a non-linear formula that can be assessed in real-time during RF ablation. AI-guided PVI has recently been associated with shorter total procedural time, higher first-pass isolation rates and significantly lower arrhythmia recurrence rates with no difference in complication rates compared to standard ablation approaches [6, 7]. However, only limited data exist on the use of AI for ablation of left atrial linear lesions. The aim of this study was to assess acute and long-term effectiveness as well as procedural feasibility and safety of AI-guided left atrial linear lesions ablation compared to CF-guided approaches.

## Methods

### Patient population

Patients undergoing catheter ablation for AF or LAT including left atrial linear lesions ablation guided by AI (AI-group) between 07/2015 and 08/2020 were prospectively included into the study. A patient cohort who underwent CF-guided ablation during the same time period without use of AI (CF-group) served as a control group. The CF-guided group represents patients treated earlier in the registry, before AI was incorporated into the mapping workflow. AI implementation at our center began in 2018 and was subsequently used for linear lesion guidance in consecutive cases, while the ablation catheter platform remained identical in both groups. The study was approved by the local ethics board (Lübeck ablation registry ethical review board number: 22–144) and carried out in compliance with the ethical principles established in the 1964 Declaration of Helsinki and its amendments. All patients provided written informed consent for the procedure and anonymized prospective data analysis.

### Preprocedural management

All patients underwent preprocedural transesophageal echocardiography to rule out intracardiac thrombi. No additional imaging was conducted prior to ablation. In patients on vitamin K antagonists periprocedural therapeutic INR values of 2–3 were aimed for. In patients treated with direct oral anticoagulants the morning dose was omitted on the day of ablation.

### General ablation approach

The detailed approach of 3D mapping and left atrial ablation including PVI and linear lesion ablation has been described before [8]. In brief, the procedure was performed in deep sedation using midazolam, fentanyl and continuous infusion of propofol. Three ultrasound-guided femoral punctures were performed and three short 8 F-sheaths were inserted. A diagnostic decapolar catheter was positioned in the coronary sinus (CS). Double transseptal puncture was performed under fluoroscopic guidance using a modified Brockenbrough technique with 8.5 F transseptal sheaths and a puncture needle (SL1 sheath and BRK-1 TSP needle, St. Jude Medical, Inc., St. Paul, MN, USA). Pulmonary vein angiographies were performed in all patients over the transseptal sheath or a 7 F multipurpose catheter. After successful transseptal puncture heparin boluses were administered targeting an activated clotting time of > 300 s. Ablation and electroanatomic mapping were performed with a contact force enabling open-irrigated tip catheter (Thermocool Smart-touch SF D or F curve, Biosense Webster, Diamond Bar, CA, USA). A spiral mapping catheter (LASSO, Biosense Webster) was used for electrical assessment of the pulmonary veins (PVs) during PVI and for left atrial pacing maneuvers as well as for creation of electroanatomical maps. Patients presenting in persistent AF were cardioverted to sinus rhythm prior to left atrial mapping. PVI was performed as wide area circumferential ablation with entrance and exit block of all PVs as the procedural endpoint. Linear lesion ablation was performed either in cases of macro-reentrant LAT or for substrate modification of low-voltage areas. According to common definitions, absence of voltage or a bipolar voltage amplitude ≤ 0.05 mV was defined as a low-voltage area. For CF-guided linear lesion ablation power settings of 40 W for anterior lesions and 30 W with an irrigation flow rate of 20 ml/min for posterior lesions were chosen aiming at 10–40 g CF, inter lesion distances < 6 mm and an ablation duration of 20–30 s per point and a minimum reduction of signal amplitude of 70%. In patients undergoing AI-guided ablation power settings were 30–50 W, aiming for an AI of 380 for posterior segments, 450 for roof segments and 550 for anterior segments with interlesion distances < 6 mm and an irrigation flow rate of 20 ml/min. In the CF cohort, 40 W was used as the initial intended power for AL and anterior endocardial MIL ablation, but power could be manually reduced at the operator’s discretion. Epicardial CS ablation was routinely delivered at 20–30 W, which is reflected in application-level power distributions.

### Anterior lines

Anterior lines (AL) were ablated between the anterior mitral annulus and the right superior pulmonary vein (RSPV). During ablation the circular mapping catheter was placed inside the LAA. Conduction block along the anterior line was validated during sinus rhythm by an abrupt prolongation of conduction time into the left atrial appendage (LAA) of at least 50 ms. AL were also checked for double potentials along the whole line with pacing at either side of the line to indicate conduction block. Furthermore, pacing from the LAA via the lasso catheter was performed with simultaneous mapping along the AL. Early signals at the sites preceding the line and late signals on the other side were indicators for conduction block. The activation sequence along the mitral annulus needed to be clockwise during LAA pacing when AL block was achieved. Pacing maneuvers were performed in opposite direction to assess for bidirectional lesion block.

### Mitral isthmus lines

Mitral isthmus lines (MIL) were ablated between the posterolateral mitral annulus and the left inferior pulmonary vein (LIPV) ostium. For assessment of conduction block the CS catheter was placed across the MIL and pacing was performed from the LAA with the circular mapping catheter. A sudden change of the activation sequence on the CS catheter was defined as complete block of MIL. If conduction block was not achieved endocardially, epicardial ablation over the coronary sinus was always performed to attempt completion of bidirectional block in both the CF and AI cohorts; no case with incomplete endocardial block was left without a CS ablation attempt.

### Roof lines

Roof lines (RFL) were ablated between the RSPV and the left superior pulmonary vein (LSPV). Conduction block along RFL was demonstrated by pacing the circular mapping catheter placed in the LAA and moving the ablation catheter at the posterior wall below the RFL towards more inferior parts of the posterior wall with local signals on the ablation catheter becoming consecutively earlier under LAA pacing. Pacing from the ablation catheter was used to demonstrate bidirectional lesion block.

### Posterior wall isolation

Posterior wall isolation (PWI) was achieved by ablation of a RFL and a linear lesion connecting the right inferior pulmonary vein (RIPV) with the LIPV resulting in a posterior box lesion. Completeness of PWI was defined as complete loss of detectable signals inside the posterior box lesion and demonstration of an exit block by pacing via the ablation catheter from inside the posterior box lesion without atrial capture. Ablation inside the box to achieve PWI was on discretion of the operators.

### Post-procedural care, follow-up and repeat ablation procedures

Transthoracic echocardiography was performed immediately following the procedure and the next day to rule out pericardial effusion. The femoral access sites were closed with a figure-of-eight suture. All patients were moved to a wake-up area. After an observation period of 2–4 h, patients were transferred to the general ward. The groin stitches were removed the next day. Preexisting DOAC therapy was continued 6 h following ablation. VKA therapy was continued, with an INR of 2.0–3.0. Proton-pump inhibitors were administered to all patients for 6 weeks following PVI. Anti-arrhythmic drug therapy was prescribed for three months following ablation. Clinical follow-up was performed at our outpatient clinic after three, 6 and 12 months, which included an assessment of the clinical history, a 12-lead ECG and a 24-hours holter ECG. Device interrogation and routine transthoracic echocardiographic controls were performed every six months. Arrhythmia recurrence was defined as any atrial arrhythmia recurrence beyond three months blanking period. In cases of arrhythmia recurrence outside the three months blanking period, repeat ablation was scheduled. Prior ablated PV lesions and left atrial linear lesions were assessed for persistence of conduction block. In cases of electrical reconduction repeat ablation was performed. Findings during repeat ablation procedures after the initial linear lesion ablation (index procedure) were included into the analysis.

### Post-procedural analysis of lesion characteristics

Data of AI-guided and CF-guided and lesions were exported from the CARTO 3 software for retrospective analysis. Every ablation tag which was recorded during ablation procedures was manually assigned to specific linear lesions. Number of applications, mean duration of applications, temperature and impedance values, force values, force-time integral (FTI) and AI values were assessed. The length of lesions was measured manually using the CARTO software.

### Statistics

Continuous variables were expressed as mean ± standard deviation or standard error of the mean for normal distributions or as median/interquartile range (IQR) for non-normal distributions. Categorial variables were summarized as counts (%). One-way analysis of variance (ANOVA) including Post-hoc Tukey testing was performed for data comparison. All analyses were performed using SPPS version 25 (IBM Cooperation, Armonk, New York).

## Results

### Baseline characteristics

A total of 313 patients (156 males, 49.8%) were included into the study. Patients in the AI-group did not differ significantly in terms of baseline characteristics from patients in the CF-group. Details on patient characteristics are summarized in Table 1.

**Table 1:** Baseline characteristics

	CF-guided ablation	AI-guided ablation	*P* value
Number of patients, n (%)	53 (16.9)	260 (83.1)	1
Male gender, n (%)	23 (43.4)	133 (51.2)	0.31
Age at procedure	70.7 ± 8.3	70.3 ± 9.3	0.74
BMI [kg/m²]	29.2 ± 5.3	27.7 ± 4.9	0.05
LVEF (%)	53.5 ± 6.9	51.3 ± 9.8	0.12
CAD, n (%)	1 (1.9)	14 (5.4)	0.42
DCM, n (%)	4 (7.6)	2 (0.8)	0.28
Other SHD, n (%)	5 (9.4)	13 (5)	0.21
CHA2DS2-VASc Score	3.36 ± 1.61	3.11 ± 1.78	0.35
OAC, n (%)	49 (92.5)	229 (88.1)	0.36
DOAC, n (%)	21 (39.6%)	156 (59.6%)	0.01
CIED, n (%)	14 (26.4)	61 (23.5)	0.92
Arrhythmia at index procedure			
PAF, n (%)	12 (22.6)	27 (10.4)	0.21
PersAF, n (%)	23 (43.4)	135 (0.5)	0.29
AT, n (%)	18 (34)	98 (37.7)	0.64

*CF c*ontact force, *AI a*blation index, *BMI b*ody mass index, *LVEF l*eft ventricular ejection fraction,* CAD c*oronary artery disease,* DCM d*ilative cardiomyopathy,* SHD s*tructural heart disease,* OAC o*ral anticoaculation,* DOAC d*irect oral anticoagulation,* CIED c*ardiac implantable electronic device,* PAF p*aroxysmal atrial fibrillation,* PersAF p*ersistent atrial fibrillation,* AT a*trial tachycardia

### Procedural characteristics

A total of 260 patients (83.1%) underwent AI-guided ablation and 53 patients (16.9%) CF-guided ablation. In the group of AI-guided ablation 213 patients (81.9%) received AL, 35 patients (13.5%) MIL, 114 patients (43.8%) RFL and 63 patients (24.2%) PWI. In the group of CF-guided ablation 47 patients (88.7%) received AL, 9 patients (17.0%) MIL, 17 patients (32.1%) RFL and three patients (5.7%) PWI. Coronary sinus epicardial ablation was required to achieve acute MIL block in 6/9 (66.7%) patients in the CF-guided arm and in 12/35 (34.3%) patients in the AI-guided arm. Periprocedural complications occurred in 22 patients (7%) with equal distribution in both CF-guided and AI-guided ablation (*p* = 0.31). Procedural related pericardial effusion without the need of intervention was the most common complication in both groups (1.9% in CF-guided ablation vs. 2.7% in AI-guided ablation, *p* = 1). Further details have been depicted in Table 2.

**Table 2:** Procedural characteristics

	CF-guided ablation	AI-guided ablation	*P* value
AL, n (%)	48 (90.6)	213 (81.9)	0.62
MIL, n (%)	9 (16.9)	35 (13.5)	0.75
RFL, n (%)	17 (32.1)	114 (43.8)	0.82
PWI, n (%)	3 (5.7)	63 (24.2)	0.14
Periprocedural complications, n (%)	2 (3.8)	20 (7.7)	0.31
Cardiac tamponade, n (%)	0 (0)	2 (0.8)	1
Pericardial effusion, n (%)	1 (1.9)	7 (2.7)	1
Retroperitoneal bleeding, n (%)	0 (0)	1 (0.38)	1
Groin hematoma, n (%)	1 (1.89)	0 (0)	0.17
Pseudoaneurysm, n (%)	0 (0)	3 (1.2)	0.67
Phrenic nerve palsy	0 (0)	1 (0.4)	1
Stroke/TIA	0 (0)	2 (0.8)	1
Permanent pacemaker implantation, n (%)	0 (0)	3 (1.2)	1

*AL a*nterior line,* MIL m*itral isthmus line,* RFL r*oof line,* PWI p*osterior wall isolation,* CF c*ontact force,* AI a*blation index

### Linear lesion characteristics

**Fig. 1:**
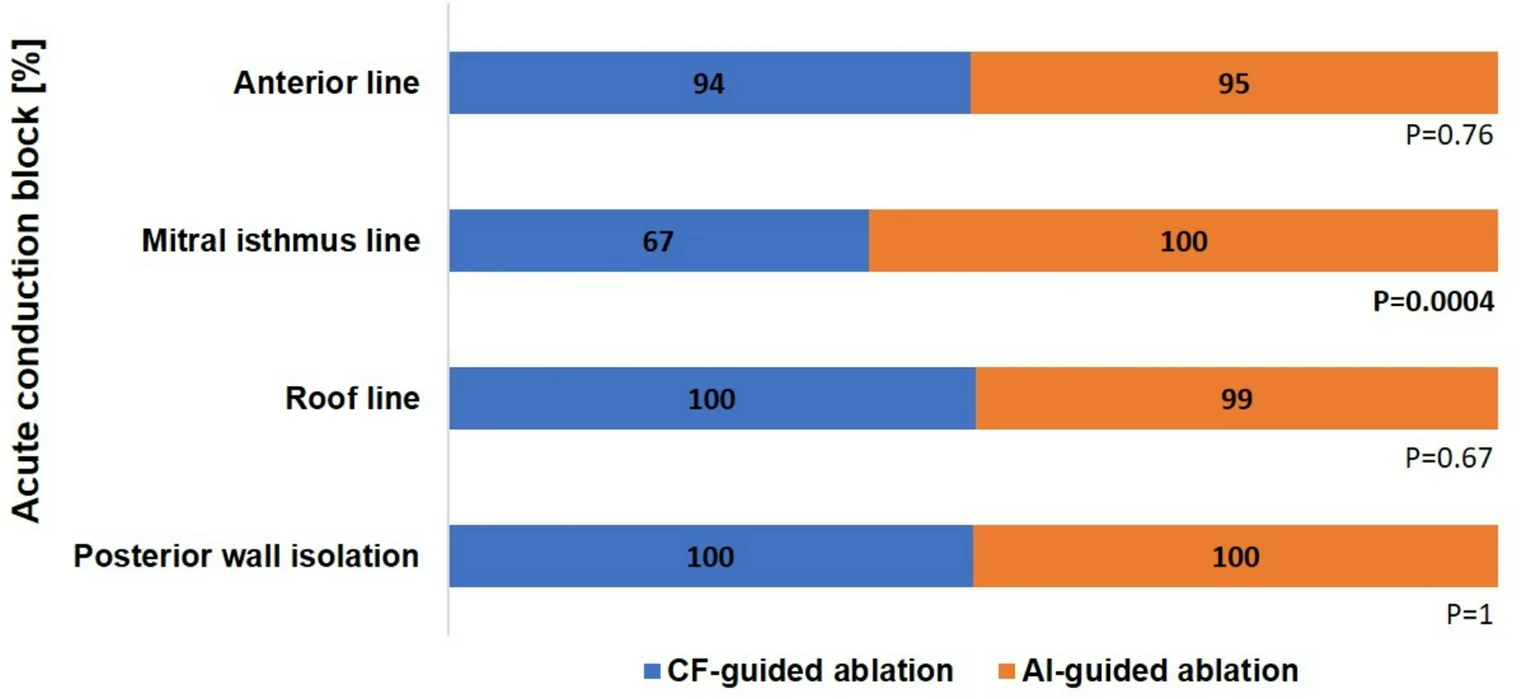
Frequency of acute conduction block across left atrial linear lines. Complete conduction block of mitral isthmus lines was significantly more often achieved when ablation index was used to guide ablation

The procedural endpoint of acute block along deployed linear lesions was achieved in the majority of patients in both study groups irrespective of the specific type of linear lesion. In the AI-group acute rates of block for AL were 94%, for MIL 67%, for RFL 100% and for PWI 100%. Rates of acute block were comparable between the two study groups for AL (*p* = 0.76), RFL (*p* = 0.67) and PWI (*p* = 1). However, acute block of MIL was achieved significantly more often in patients undergoing AI-guided ablation compared to CF-guided ablation (35/35 (100%) patients vs. 6/9 (67.7%) patients, *p* = 0.004; Fig. 1). Mean length of linear lesions, mean RF time per linear lesion, the number of RF applications per linear lesion and the average percentage of impedance drop per application were comparable between the AI-guided and CF-guided group (Table 3). Significantly higher maximum power levels were achieved in patients undergoing AI-guided ablation of AL (37.01 ± 5.30 W vs. 30.66 ± 4.12 W, *p* < 0.0001) and RFL (32.52 ± 4.12 W vs. 25.82 ± 3.99 W, *p* = 0.0003) compared to patients who underwent CF-guided ablation. There were numerically shorter RF applications when AI-guided ablation was performed with significantly shorter RF applications for RFL (17.1 ± 6.7 s vs. 21.0 ± 7.6 s, *p* = 0.0299) and a statistical trend towards shorter applications for AL in AI-guided patients (21.4 ± 5.4 s vs. 23.1 ± 6.9 s, *p* = 0.057). Mean CF values did not differ among groups and lesions except for higher average CF in AI-guided AL ablation (28.4 ± 7.7 g vs. 25.0 ± 6.4, *p* = 0.0042). FTI values were significantly lower for AI-guided MIL and RFL as compared to CF-guided lesions (349.3 ± 187.4 vs. 504.7 ± 209.5, *p* = 0.0359 and 444.5 ± 216.8 vs. 604.2 ± 276.2, *p* = 0.0072, respectively). (Table 4)

**Table 3:** Characteristics of linear lesions

	AI-guided	CF-guided	*P*-value
Length of linear lesion [mm]			
AL	61.6 ± 9.5	61.2 ± 5.5	0.7724
MIL	32.2 ± 10.3	36.5 ± 10.2	0.2615
RFL	15.5 ± 5.7	17.3 ± 4.8	0.2049
PWI	45.6 ± 13.6	43.8 ± 10.2	0.8295
RF time per linear lesion [sec]			
AL	367.95 ± 146.85	371.72 ± 133.05	0.87
MIL	327.37 ± 270.62	290.25 ± 171.89	0.63
RFL	85.39 ± 49.93	100.93 ± 69.80	0.26
PWI	249.19 ± 125.10	230.62 ± 53.74	0.8
RF applications per linear lesion, n			
AL	17.2 ± 5.5	16.8 ± 7.1	0.6524
MIL	17.5 ± 15.8	13.6 ± 5.8	0.4683
RFL	5.1 ± 2.3	4.8 ± 2.8	0.6145
PWI	14.0 ± 5.3	11.0 ± 1.7	0.3442
Mean duration per application [sec]			
AL	21.4 ± 5.4	23.1 ± 6.9	0.057
MIL	19.1 ± 6.3	21.2 ± 7.9	0.4041
RFL	17.1 ± 6.7	21.0 ± 7.6	0.0299
PWI	17.7 ± 4.7	21.3 ± 6.1	0.2141
Mean impedance drop per application [%]			
AL	9.10 ± 2.41	8.88 ± 3.06	0.59
MIL	9.09 ± 3.44	11.52 ± 8.0	0.17
RFL	7.52 ± 2.71	7.61 ± 2.35	0.9
PWI	6.89 ± 2.20	8.29 ± 0.41	0.28
Max Power per application [W]			
AL	37.01 ± 5.30	30.66 ± 4.12	< 0.0001
MIL	34.23 ± 6.80	30.4 ± 4.32	0.11
RFL	32.52 ± 4.12	25.82 ± 3.99	0.0003
PWI	29.24 ± 3.92	25.01 ± 5.60	0.17
Mean CF per application [g]			
AL	28.4 ± 7.7	25.0 ± 6.4	0.0042
MIL	18.4 ± 7.6	17.9 ± 7.7	0.8545
RFL	28.8 ± 13.0	28.2 ± 7.7	0.8515
PWI	22.8 ± 7.0	23.0 ± 14.5	0.9579
Mean FTI per application [g*sec]			
AL	540.6 ± 150.3	547.5 ± 215.9	0.7929
MIL	349.3 ± 187.4	504.7 ± 209.5	0.0359
RFL	444.5 ± 216.8	604.2 ± 276.2	0.0072
PWI	369.2 ± 144.0	481.9 ± 269.2	0.2068
Mean AI per application			
AL	515.0 ± 41.7	n.d.	
MIL	416.0 ± 80.1	n.d.	
RFL	431.6 ± 39.5	n.d.	
PWI	395.3 ± 36.6	n.d.	

*AL* anterior line,* MIL *mitral isthmus line,* RFL *roof line,* PWI *posterior wall isolation,* CF *contact force,* AI *ablation index,* RF *radiofrequency,* FTI *force time integral

**Table 4:** Follow-up data

	CF-guided ablation	AI-guided ablation	*P* value
Patients with follow-up, n (%)	45 (84.9)	220 (84.6)	0.95
Follow-up duration [days]	1197 ± 679	756 ± 429	< 0.0001
Patients with arrhythmia recurrence, n (%)	34 (64.2)	148 (56.9)	0.28
PAF, n (%)	3 (8.8)	20 (13.5)	0.59
PersAF, n (%)	10 (29.4)	50 (33.8)	0.94
AT, n (%)	21(61.8)	78 (52.7)	0.16
Patients with repeat ablation, n (%)	19 (35.8)	85 (32.7)	0.65
Remapped AL, n	17	74	
Persistent conduction block AL, n (%)	9 (52.9)	32 (43.2)	0.47
Remapped MIL, n	2	15	
Persistent conduction block MIL, n (%)	1 (50)	11 (73.3)	0.51
Remapped RFL, n	8	35	
Persistent conduction block RFL, n (%)	7 (87.5)	22 (62.9)	0.18
Remapped PWI, n	1	20	
Persistent conduction block PWI, n (%)	1 (100)	14 (70)	1

*CF *contact force,* AI *ablation index,* PAF *paroxysmal atrial fibrillation,* PersAF *persistent atrial fibrillation,* AT *atrial tachycardia,* AL* anterior line,* MIL *mitral isthmus line,* RFL *roof line,* PWI *posterior wall isolation

### Follow-up and repeat ablation procedures

**Fig. 2:**
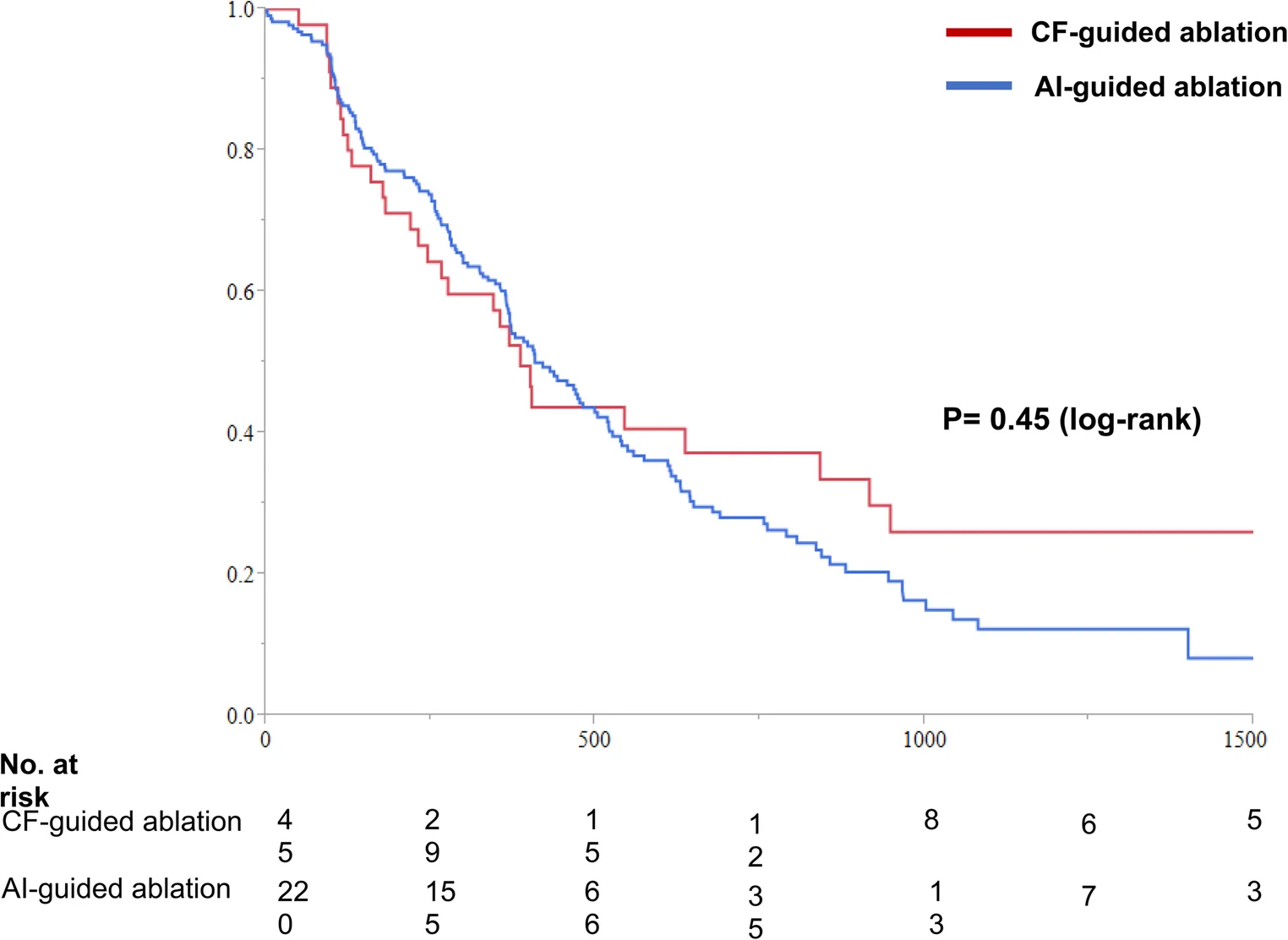
Arrhythmia free survival after linear lesion ablation. Kaplan-Meier analysis was performed for estimation of arrhythmia free survival after left atrial linear lesion ablation with and without the use of ablation index. No significant difference was observed between the two study groups regarding arrhythmia free survival

**Fig. 3:**
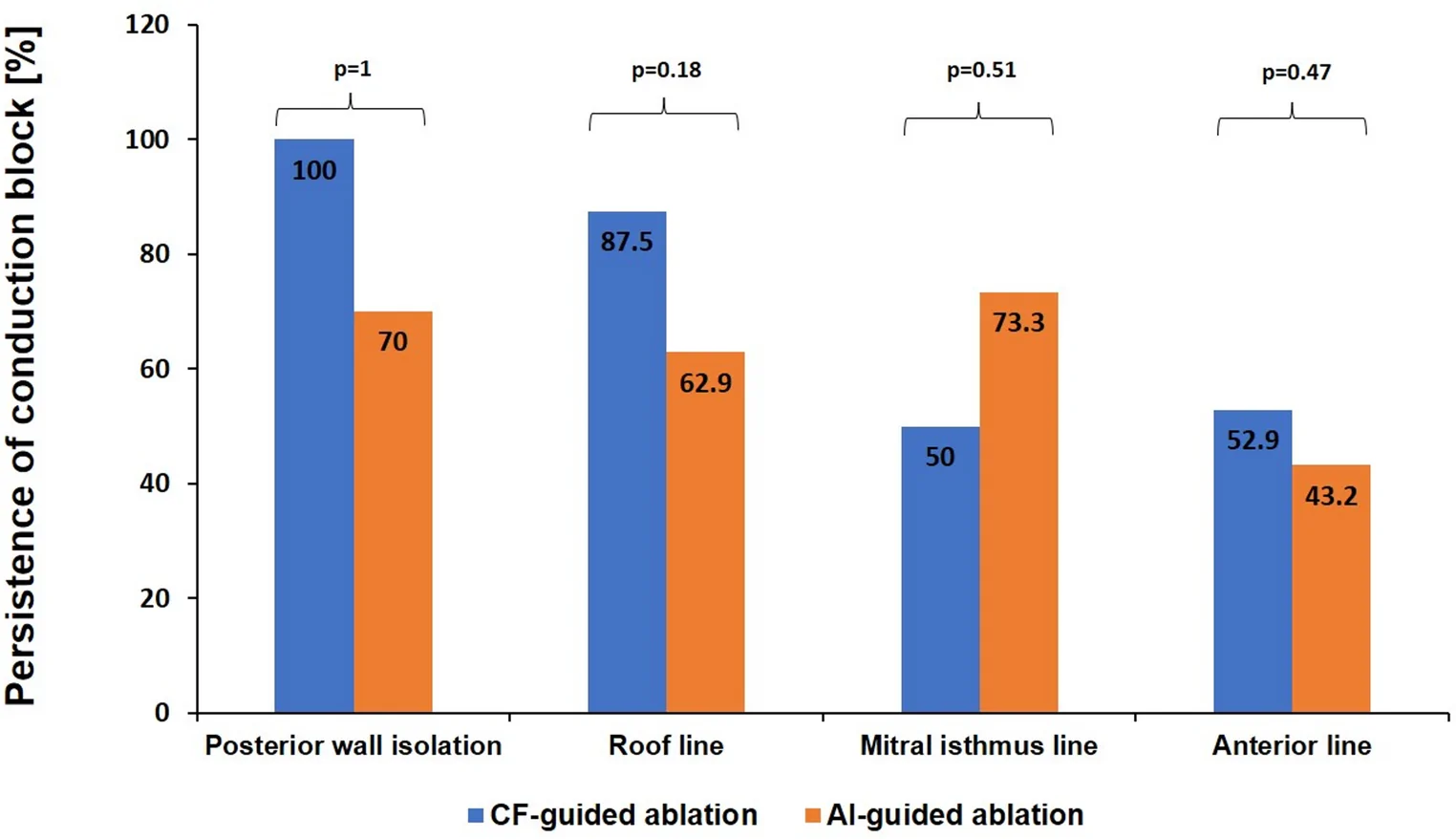
Persistence of conduction block. Rates of persistent complete conduction block across left atrial linear lesions are depicted. There was no significant difference in electrical reconduction observed between patients who underwent ablation index-guided ablation or contact force-guided ablation

Follow-up was performed in 84.9% of the patients receiving CF-guided ablation and in 84.6% of the patients receiving AI-guided ablation (*p* = 0.96) with a mean duration of 756 ± 429 days in the AI-group and 1197 ± 679 days in the CF-group (*p* < 0.0001), respectively. Arrhythmia recurrence occurred with comparable frequencies in both groups (148 patients (56.9%) of the AI-group and 34 patients (64.2%) of the CF-group, *p* = 0.28). Survival analysis showed no significant differences in estimated arrhythmia free survival (Fig. 2, log rank *p* = 0.45). The dominant mode of arrhythmia recurrence was organized AT in both cohorts (52.7% for AI-guided ablation and 61.8% for CF-guided ablation, *p* = 0.16). Repeat ablation procedures were conducted in 32.7% (AI-group) and 35.8% (CF-group) of patients with arrhythmia recurrence. Persistence of conduction block was seen in the AI-group in 52.9% for AL, 62.9% for RFL, 73.3% for MIL and 70% for PWI. There were no significant differences in persistence of linear lesion conduction block for AL (*p* = 0.47), MIL (*p* = 0.57), RFL (*p* = 0.18) or PWI (*p* = 1) in the CF-group (Fig. 3).

## Discussion

To the best of our knowledge, this is the first study systematically assessing the acute and long-term efficacy as well as procedural feasibility and safety of AI-guided versus CF-guided left atrial linear lesions ablation, providing detailed insights into procedural parameters of AL, MIL, RFL and PWI. The main findings of our study are:

1. *Acute conduction block of AL*,* MIL*,* RFL*,* and PWI using either AI-guided or CF-guided ablation can be achieved in the majority of patients. AI-guided ablation is however associated with a significant higher rate of acute MIL blockage.*
2. *AI-guided ablation of MIL and RFL has significantly shorter RF application times and lower FTI values than CF-guided ablation indicating effective lesion formation.*
3. *Persistence of conduction block along linear lesions during repeat mapping was similar for AI- and CF-guided ablation. Long-term arrhythmia recurrence rates were comparable for AI- and CF-guided linear lesion ablation.*

### Acute conduction block of linear lesions

Bidirectional conduction block is the primary procedural endpoint of left atrial linear lesions ablation and mandatory to establish antiarrhythmic effects and avoid proarrhythmic states. However, achieving complete conduction block during RF ablation can be challenging. Previously published studies report acute conduction block rates for AL of 80.6–100% [9–11] with acute reconduction rates of up to 20% in non-AI-guided ablation [11], for RFL of 81–96% [12, 13] and for PWI around 86.5–94.3% [14, 15]. Wenzel et al. described the creation of AL with pulsed field ablation which resulted in acute bidirectional block in 84% [16]. Rates of acute block in our study were in line to the above mentioned previously published data. We found high rates of acute complete block for AL both in AI-guided (95%) and non-AI-guided (94%) ablation (*p* = 0.76). Complete PWI was achieved in 100% of the patients regardless of AI implementation. We also observed high rates of complete acute block of RFL (99% AI-group, 100% CF-group, *p* = 0.67). Ablation of MIL is especially challenging due to known anatomic challenges with reported rates of acute complete conduction block around 80.6–98.2% [9–11]. Notably, we found significantly higher acute conduction block rates for MIL ablation in AI-guided ablation (100%) compared to non-AI-guided ablation (67%, *p* < 0.0001). Although the 100% acute block rate of MIL in the AI cohort exceeds many prior reports, this reflects strict bidirectional conduction validation and consistent rescue epicardial ablation over the CS whenever needed, a strategy not uniformly enforced in earlier non-AI protocols. This finding underlines the potential benefit of AI-guided ablation of MIL. Numerical persistence advantages of CF-guided AL or RFL may relate to cohort differences in underlying low-voltage burden and shorter myocardial spans. AI-guided MIL showed superiority likely due to consistent CS rescue ablation and improved non-linear energy transfer optimization.

Left atrial wall thickness and intramural fat heterogeneity vary markedly across patients. These factors may limit transmurality in complex segments like the MIL where fat interposition and epicardial vessel adjacency are prevalent. Although our line lengths did not differ significantly between cohorts, AI’s non-linear integration of contact and stability may partly offset anatomical barriers. MIL efficacy nevertheless benefits from consistent CS rescue ablation to overcome intramural fat-related conduction insulation.

### Long-term persistence of conduction block and arrhythmia recurrence

Data on invasively assessed conduction recovery across left atrial linear lesions is scarce. Previously published data on invasive follow-up after linear lesion ablation observed reconduction rates of 28–79% for RFL [17–19], 24–90% for MIL [18;20] and 38.2–58% for AL [20, 21]. In our study repeat ablation procedures were conducted in 35.8% (CF-group) and in 32.7% (AI-group) of our patients with arrhythmia recurrence. We observed reconduction rates in the AI-group of 56.8% for AL, 37.1% for RFL, 26.7% for MIL and 30% for PWI. However, reconduction rates of each linear lesion did not differ significantly between patients who underwent AI-guided or non-AI-guided ablation.

Our data are in line with previously reported studies since we found relatively high rates of electrical reconduction despite initially successful conduction block across linear lesions. Reasons are thought to be of complex origin. At first, acute conduction block after deployment of a lesion cannot be achieved in all patients resulting in a need of further ablation lesions to achieve bidirectional lesion block [11, 22]. Post-procedural reconduction may occur when edema and inflammation resolve after such extensive ablation procedures. This is especially true for more challenging lesions such as AL and MIL. For AL a greater anatomical distance of myocardium with relatively variable myocardial thickness must be covered. Previous studies deployed lesions of around 60–65 mm for AL which is in line with the data from our analysis [11, 22]. For MIL anatomical neighborhood of epicardial vessels as well as relatively rare presence of marked myocardial scar make effective lesion formation challenging.

### Impact of AI on effective lesion formation

AI is a measurement tool allowing to objectively and operator-independently predict quality of lesion formation during catheter ablation. Reference values have been published for ablation of left atrial arrhythmias [8, 11] and AI has been routinely implemented in ablation protocols for PVI and other ablation approaches [22, 23]. There are only few analyses of AI-guided ablation for left atrial linear lesion creation [11, 22]. We found that AI-guided linear lesion creation was similar effective compared to standard ablation with CF-sensing catheters for creation of AL, RFL and PWI and was more effective for creation of MIL. RF applications were shorter when AI-guided ablation was utilized and FTI values were lower. Our findings are in line with a previous study of Santoro et al. that found shorter RF applications for AI-guided ablation as compared to standard ablation with CF catheters [11]. In contrast to our observation AI-guided ablation was performed with a smaller number of RF applications in this analysis [11]. We found no difference in total ablation times and average numbers of RF applications but AI-guided ablation had numerically more applications when analyzed individually for each type of linear lesion. In a study by Zanchi et al. AI-guided ablation of AL and RFL using a high-power short-duration (HPSD) protocol utilizing 50 W with targeted AI values of 550 anterior and 400 posterior was studied [22]. Zanchi et al. found a comparable high rate of intraprocedural lesion block (AL 97%, RFL 100%). Of note, the use of HPSD throughout all procedures resulted in short ablation times for both AL and RFL with an of average 10.0 s per lesion for AL and 7.3 s for RFL [22]. As compared to the study by Zanchi et al. we observed longer RF applications in our analysis with average duration of RF application of 17 to 21 s for all types of linear lesions. In our study cohort a more conservative approach without the standard use of HPSD was utilized for ablation of linear lesions thereby explaining longer durations of RF applications as compared to above mentioned study.

AI-guided ablation may result in more effective lesion formation as compared to standard ablation through several mechanisms. When modern indices such as AI are not utilized operators guide ablation by other surrogates like signal amplitude reduction, impedance drop or FTI. It has been described before that these parameters are less reliable in clinical practice than lesion indices [8]. A previous study from our group found that AI values are more robust with smaller variability between RF applications as compared to FTI when it is incorporated into ablation approaches aiming at PVI [8]. FTI increases linear with the duration of RF application and does not incorporate other factors such as tissue contact, power or respiration-mediated catheter instability. In contrast, AI increases not linear reflecting a more realistic energy transfer to ablated tissue. In clinical practice operators who rely on AI can adapt their ablation approach to circumstances such as tissue contact and catheter stability. The result may be shorter ablation applications, less edema formation and finally more effective lesion creation. Additionally, use of AI may be beneficial in terms of periprocedural safety and the frequency of periprocedural adverse events was not different between AI-guided ablation and standard ablation indication feasibility of this approach.

### Limitations

The study has several limitations. First, it was a single-center non-randomized study with a limited number of patients. Larger cohorts are needed to validate these data. Second, only a limited number of patients with arrythmia recurrence underwent repeat ablation. The frequency of blocked lines may be biased and would have been higher or lower if all patients underwent repeat invasive mapping. Third, the follow-up duration varied across patients who underwent AI-guided or CF-guided ablation which makes data on arrhythmia recurrence less comparable. Additionally, power settings differed between operators and were not fixed across cohorts; therefore, the study was not a power-equivalency comparison and RF power differences could confound procedural efficiency outcomes.

## Conclusions

Acute conduction block for left atrial linear lesions can be achieved in the majority of patients both by AI-guided and non-AI-guided ablation. AI-guided ablation is however associated with significantly higher rates of acute conduction block across MIL and shorter RF times and lower FTI for AL and RFL while complication rates are comparable to non-AI-guided ablation.

## Data Availability

No datasets were generated or analysed during the current study.
